# Lung cancer care pathways and journeys: insights from patients at the National Cancer Institute in Mexico

**DOI:** 10.1186/s44263-026-00278-7

**Published:** 2026-05-27

**Authors:** Elysse Bautista-González, Cecilia Vindrola-Padros, Alejandro Mohar Betancourt, Alberto Monroy-Chargoy, Heber Tomás Reyes-García, Hynek Pikhart, Anne Peasey

**Affiliations:** 1 Institute of Epidemiology and Health Care, University College London, London, UK; 2 Department of Targeted Intervention, University College London, London, UK; 3 Instituto de Investigaciones Biomedicas, UNAM, Instituto National de Cancerologia, Ciudad de Mexico, Mexico; 4 Hospital General de León, León, México; 5 Centro Médico Nacional 20 de Noviembre, Ciudad de México, México

**Keywords:** Lung cancer, Patient journey, Mexico, Mixed methods

## Abstract

**Background:**

Delayed diagnosis and treatment remain major contributors to poor lung cancer outcomes in Mexico, where fragmented service delivery and weak referral coordination shape patient trajectories. Integrating patient narratives with measurable time intervals is critical to understanding how health system structures translate into lived delays and inequities.

**Methods:**

We conducted a convergent mixed-methods study using routinely collected electronic health record data and structured patient interviews at the National Cancer Institute of Mexico (INCAN). Relevant dates were extracted for all suspected lung cancer patients admitted between 2004 and 2021 (*N* = 2,645). Descriptive statistics, interval calculations, pathway classification, and temporal trends were drawn. In parallel, a purposive subsample of patients (*N* = 46) participated in structured interviews exploring symptom appraisal, help-seeking, travel, and interactions with health services. Quantitative and qualitative data were collected concurrently and analysed independently with equal priority. Open-ended interview data were analysed thematically, and structured electronic health record data were quantified. Integration then occurred through joint displays combining interview-derived data with medical record variables to link measured delays and clinical pathways with patients’ contextual characteristics and reconstruct individual care journeys.

**Results:**

Four diagnostic pathways were identified, reflecting where and how diagnosis occurred in *N* = 2645 patients. Median time from first symptom to treatment ranged from 160 to 255 days, with substantial variation by pathway and year of admission. Interviews revealed *N* = 41 patients were symptomatic at the time of first appraisal. Recurrent patterns of delayed self-appraisal (*N* = 61 (median days to first encounter after first symptom), misdiagnosis (*N* = 24), fragmented referrals, financial insecurity, and long-distance travel to access specialised care. Patients commonly navigated multiple public and private providers before reaching INCAN with very few arriving solely from a public pathway, reaching a median of 3 medical encounters before diagnosis, and thus often incurring out-of-pocket costs and experiencing interruptions in care.

**Conclusions:**

Stratifying lung cancer outcomes by diagnostic pathway and linking interval-based metrics to patient experience reveals how health system fragmentation generates avoidable delays and inequities. Policy responses should prioritise coordinated referral pathways, strengthened regional diagnostic capacity, financial protection mechanisms, and early detection competencies across levels of care.

**Supplementary Information:**

The online version contains supplementary material available at 10.1186/s44263-026-00278-7.

## Background

Cancer care pathways are biomedical tools that mark key clinical activities and goals along the continuum of care to improve continuity and outcomes [1, 2].They delineate events and intervals that are important for timely diagnosis and treatment [2–5]. Frameworks such as those of Andersen et al. and Walter et al. describe both the patient- and system-level processes that influence these intervals, situating them within the dimension of time [6–8]. Measuring time interval outcomes allows for delays to be compared across different populations, identifying patterns and enriching the evidence base for cancer care improvement [9].

Unlike pathways, journeys are patient-centred, dynamic, and often unpredictable, shaped by the interplay of individual, social, and system-level factors. Studying people’s narratives and promoting their dissemination raises awareness of the challenges patients face [10, 11], which may lead to late cancer care, higher costs, and poorer outcomes [6, 7, 12, 13]. Understanding the journeys people face humanises research, reminding clinicians, academics, and policymakers of the individuals behind the statistics, and can ultimately contribute to system-level change [10, 11].

Studying pathways and journeys is especially relevant for lung cancer care in Mexico, where it accounts for roughly 10% of all cancer deaths [14]; diagnosis and treatment are frequently delayed [15, 16]; and the health system is fragmented [17–19]. Evolving around occupation-specific institutions rather than a cohesive, unified health system [17–19], each one of the health care delivery institutions has a different funding scheme. Patients are restricted to services within affiliated institutional premises [17–19], preventing patients from navigating the cancer care freely and instead enduring tortuous journeys that lead to delays in care. Additionally, there are disparities in per capita health spending between these delivery institutions, giving rise to pronounced health inequalities [20]. For patients with lung cancer particularly [20], this might result in differences in access to treatments and catastrophic expenditures [20, 21].

We contribute to the literature by examining the patients’ *healthscapes* (the collective set of interactions and potential care transitions and treatment options that patients envision, contribute to, and navigate) [1, 22], through a mixed-methods approach. This encompasses studying both, pathways (system structures, institutional routes) and journeys (lived experiences). In doing so, the study provides a comprehensive understanding of lung cancer patient challenges and identifies opportunities for research, intervention, and policy improvement in Mexico.

## Methods

### Study design

As part of a larger research programme [23], this convergent mixed-methods study triangulated routinely collected health record data with patient semi-structured interview data. The quantitative component assessed time intervals, referral pathways, and patterns of care across a large cohort, while the qualitative component contextualised how and why these pathways unfolded from the patient perspective. The mixed-methods approach was used to link measured delays and pathways to lived experience. Quantitative data were extracted from electronic health records for all eligible patients admitted to the National Cancer Institute of Mexico (INCAN) between 2004 and 2021 (*N* = 2645). Qualitative data were collected through structured interviews with a purposive subsample of patients (*N* = 46).

### Eligibility criteria

Two samples were included: (1) The retrospective chart review included patients aged 18 years or older with a primary diagnosis of lung cancer admitted to INCAN between 2004 and 2021, and (2) Structured interviews included patients aged 18 years or older with lung cancer admitted between June 2019 and March 2021. Patients with poor performance status who were too unwell to participate were excluded (Eastern Cooperative Oncology Group Performance status ECOG ≥ 4) [24].

### Recruitment

Interview participants were purposively selected from recent admissions to ensure diversity of care experiences. To capture both pre-COVID-19 and pandemic journey experiences, patients admitted up to 12 months before June 2020 were eligible for interview. Participants were approached between admission and treatment completion. All participants received written study information and provided written informed consent. No refusals were recorded. Participants had no prior relationship with the interviewer and were recruited solely through study invitation.

### Data collection

#### Electronic health records

Health record data included sex, age, relevant interval dates defined using the Aarhus Statement [8], diagnosis site, and treatment received. Data were extracted for patients admitted between 2004 and 2021. Detailed extraction procedures for dates have been previously published [15].

#### Interviews

Supplementary Material 1 includes the interview guide developed prior to data collection. It was informed by Levesque et al.’s access-to-care framework [25] and previous literature from Mexico and the Aarhus statement [26, 27].These frameworks were used to structure the guide around broad dimensions of access. The guide was semi-structured, which allowed for consistent coverage of access dimensions while permitting participants to narrate their experiences in their own terms. For example, closed questions captured a list of first symptoms, key dates, first healthcare contacts, travel time, and transport to INCAN. Open-ended questions elicited free narrative accounts of the patient pathway from symptom onset to admission. The interview guide was reviewed within the research team. Minor refinements were made during early interviews to improve clarity and flow, without altering core domains.

Structured interviews were conducted in Spanish between June 2020 and March 2021 by EBG, a PhD student at the time of data collection. Participants were informed that the interviewer was a researcher and had no prior clinical relationship with them. Interviews took place in private rooms within outpatient areas. Family members or caregivers were sometimes present, depending on patient preference. Each interview lasted approximately one hour. Interviews were audio-recorded. Structured responses were documented during the interview, and open-ended questions relied primarily on audio recordings, supported by field notes when clarification was required. No repeat interviews were conducted. Audio recordings were transcribed in Spanish by EBG. Transcripts were not returned to participants for correction.

**Table 1:** Overview of the mixed-methods study design and integration

Component	Quantitative	Qualitative	Mixed methods (integration)
**Data source**	Electronic health records from INCAN	Structured patient interviews	Linked health record and interview data
**Sample size**	*N* = 2645	*N* = 46	*N* = 46 (interview subsample linked to records)
**Data collection**	Retrospective extraction of routinely collected clinical data (2004–2021)	Audio-recorded structured interviews conducted in Spanish (2020–2021)	Concurrent collection; interview sampling informed by quantitative pathways
**Type of data**	Numeric, date-based, categorical	Narrative, experiential, contextual	Combined numeric timelines and narrative accounts
**Key variables / content**	Sex, age, diagnosis site, treatment, Aarhus-defined intervals, care pathway	Symptom appraisal, help-seeking, transport, travel time, actors visited, etc.	Intervals, entry pathway, sex, transport, travel burden, number of actors, narrative explanations
**Analytic approach**	Descriptive statistics, interval calculations, pathway classification, temporal trends	thematic analysis using Braun and Clarke’s framework	Displays aligning timelines with narratives; journey reconstruction
**Software**	Stata statistical software: Release 18. StataCorp LLC, College Station, TX, USA	NVivo (Version 14). Lumivero, Denver, Colorado, USA	joint-display matrices
**Primary analytic focus**	Pathway-level patterns and system-level performance	Contextualising individual patient experience	Linking measured delays to lived experience
**Role in the study**	Defines pathways, intervals (delays), and trends over time	Explains how and why delays occur	Integrates delays and experience
**Ultimate output**	Quantified delays, pathway typology, trends by year.	Themes describing barriers, in the lung cancer journey in Mexico.	Policy-relevant interpretation

## Analysis

Quantitative and qualitative data were collected concurrently and analysed independently, with equal analytic priority. Qualitative thematic analysis and quantification of interview data were completed prior to mixed-methods integration. Table 1 describes the parallel quantitative and qualitative components of the study and how they were integrated.

### Quantitative

#### Interval trends

For patients who received treatment, the total interval from first symptom to treatment initiation was calculated. Intervals were compared across calendar years and care pathway groups to assess variation between 2004 and 2021. Pathway-level patterns and system-level performance over time were derived exclusively from the INCAN electronic health records dataset.

#### Pathway diagram

A patient flow diagram summarized care pathways across all patients (*N* = 2645), including the interview subsample (*N* = 46). Interview sampling was informed by care pathway categories identified in the health record data. Recruitment continued until sufficient representation across pathway types was achieved and patient accounts became repetitive, with no new pathway-specific experiences emerging [28, 29]. Then calculation of their time to events was conducted for each group.

### Qualitative

#### Thematic analysis and journey diagrams

Qualitative analysis followed a deductive–inductive thematic approach. Levesque et al.’s framework [25] informed the initial analytic domains. Within these domains, transcripts were analysed inductively. Codes were generated from participants’ accounts rather than pre-specified in detail. Coding was iterative and conducted by EBG. Supplementary Material 2: Coding book was refined as analysis progressed, and earlier transcripts were revisited. NVivo software was used to support data management and coding.

Thematic analysis followed Braun & Clarke’s framework [30]. Themes captured patterns related to barriers, interpretations, encounters, and system navigation. Interpretation was informed by pathway typologies and relevant literature [31–34]. Verbatim excerpts and journey diagrams are used to illustrate findings using *Preceden timeline maker* (www.preceden.com).

Selected qualitative data were quantified to support mixed-methods integration. This included counts of actors consulted, transport modalities, and care-seeking sequences derived from interview responses. Quantification was used to characterise journey features and align patient-reported experiences with clinical timelines.

### Mixed methods

#### Joint-display and triangulation

In accordance to the literature, joint displays were constructed after completion of both quantitative analyses and qualitative analyses [35]. Joint displays combined interview-derived data (transport modality, travel time to INCAN, number of actors consulted, initial diagnoses) with medical records (time-to-event intervals, entry pathway, sex). Integration supported alignment of patient-reported experiences with clinical timelines and enabled reconstruction of individual care journeys.

Patient narratives were consistent with medical records and sociodemographic data, and no substantive discordance between data sources was identified. When duplicate date information was found, dates from medical records were prioritised over interview provided data (see Table 2).

#### Researchers’ characteristics and reflexivity

EBG conducted all interviews and initial analyses. Her medical training supported rapport-building and contextual understanding of clinical pathways. CVP, a qualitative researcher and anthropologist with expertise in health systems and rapid analysis, supervised the qualitative component. Reflexive discussions between EBG and CVP were held throughout the analysis to examine assumptions and analytic decisions. The analytic stance focused on health services research rather than anthropological interpretation.

This study is reported in accordance with the Consolidated Criteria for Reporting Qualitative Research (COREQ) checklist [36] attached in Supplementary Material 3: COREQ Checklist and served a contextualising function, informing interpretation of quantified delays without generating pathway-level classifications.

## Results

### Pathway-level patterns and system-level trends

**Fig. 1:**
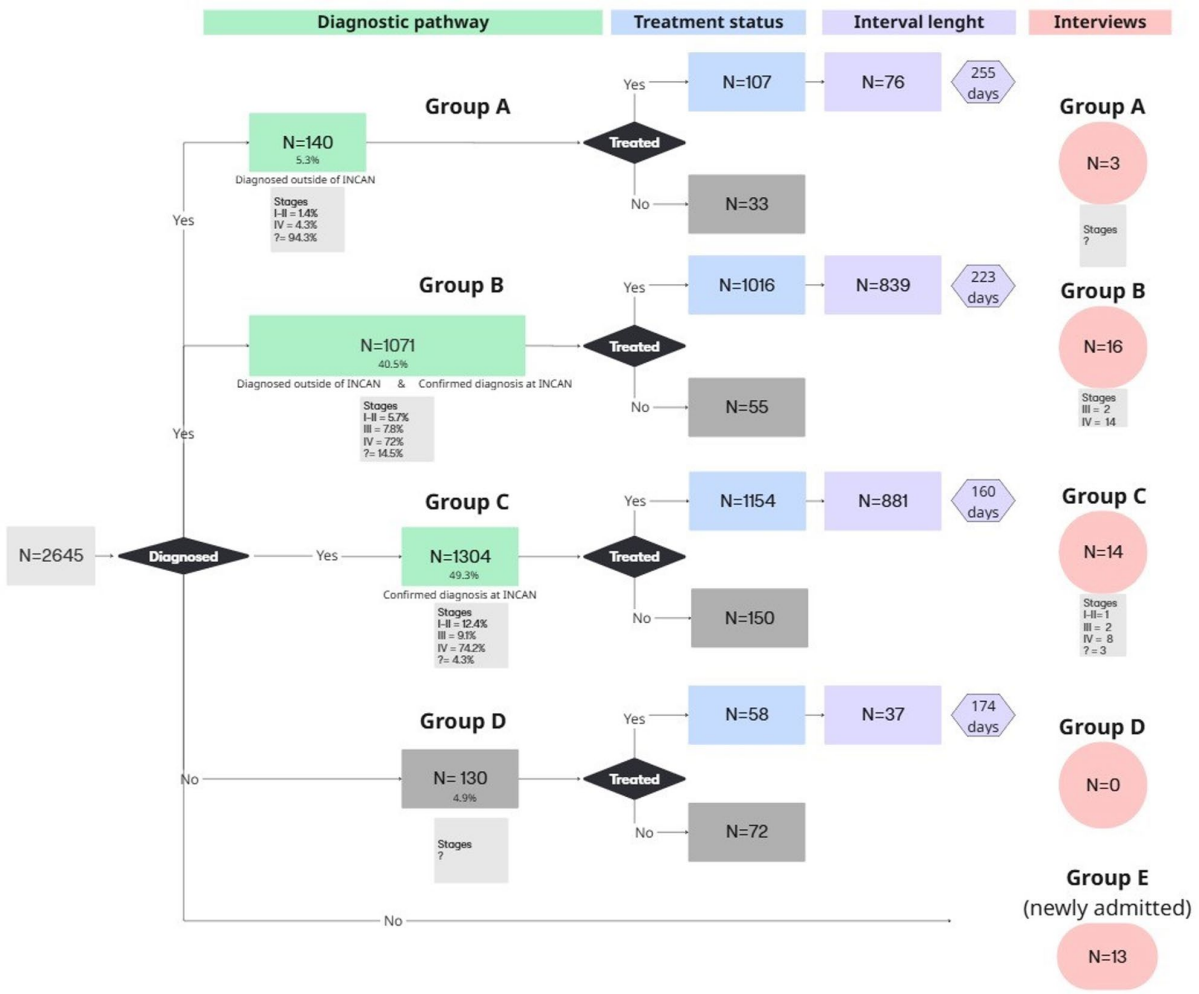
Patient flow across the pathway to lung cancer treatment. INCAN: National Cancer Institute of Mexico. Cancer stages in Roman numbers according to AJCC TNM (8th edition) [59]. Original patient sample: 2645. There are 5 different types of patients (A-E). Pink circles represent the people interviewed in each group. Group E: Patients interviewed right after admission that were not linked to the electronic health record dataset. Median total time intervals of each group are shown in purple. Missing cancer stages are marked with a question mark

From the original sample of patients for whom electronic record data was recorded (*N* = 2,645), 5% of patients were not diagnosed after admission, 5.3% were diagnosed prior to entering INCAN, 40.5% received diagnoses both at INCAN and at another institution, and 49.3% were diagnosed at INCAN only. This resulted in four care pathways (A–D), marked in green in Figure 1.

From each care pathway, the subsample of participants interviewed most frequently belonged to groups B, C, and the undetermined group E (marked in pink in Figure 1). Group E represented patients who were interviewed right after admission and thus were not linked to the electronic health records dataset. Therefore, none of the clinical variables like cancer stage were collected or intervals calculated nor linked to the interview dataset.

**Fig. 2:**
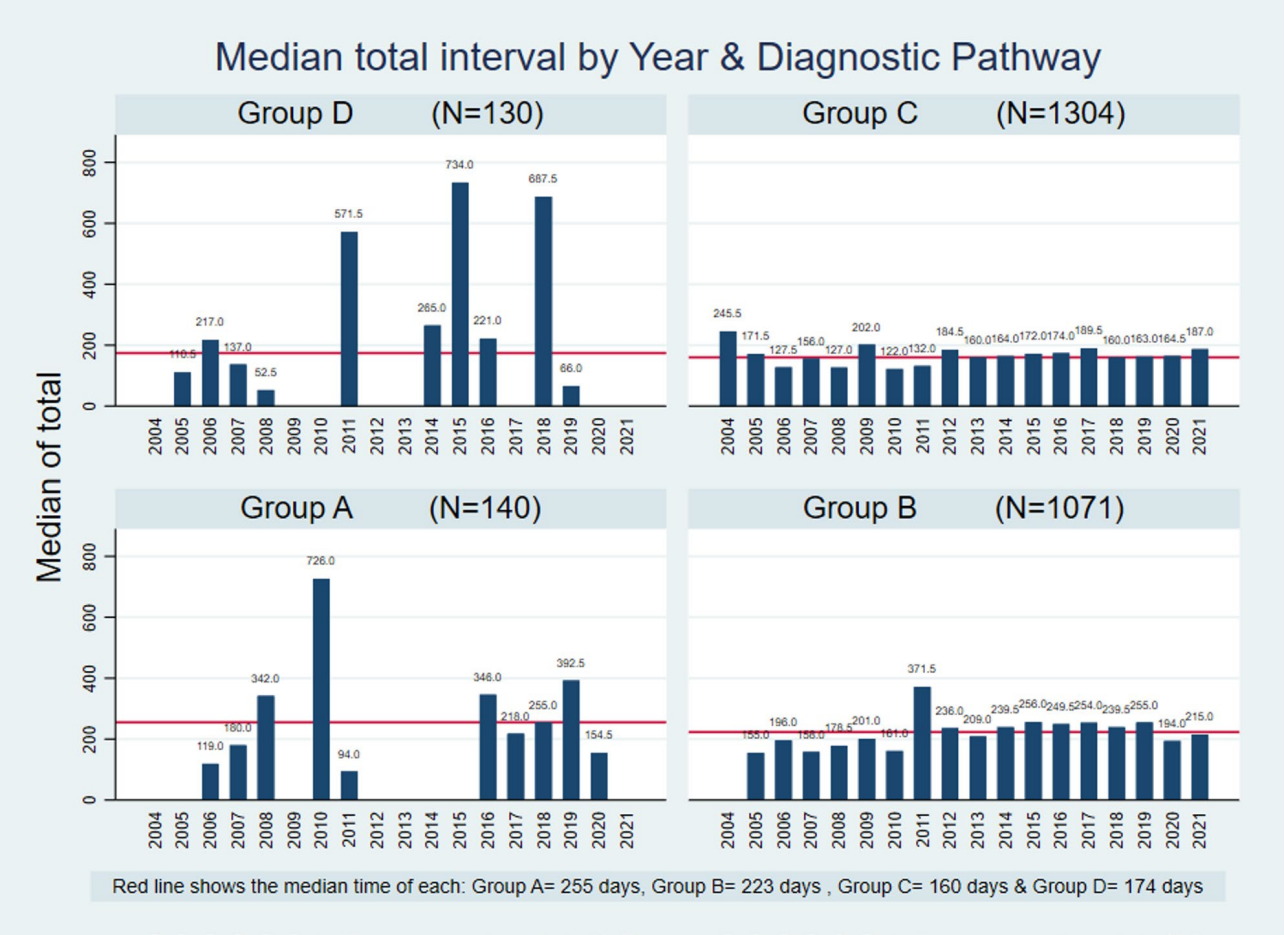
Median total interval in the lung cancer sample studied at the INCAN from 2004 to 2021 according to diagnostic pathway (*N* = 2645)

In each pathway, some patients received treatment while others did not. For those who were treated, the median time from first symptom to treatment (total internal marked in purple in Figure 1) ranged from 160 to 255 days with a median of 192 days. Figure 2 shows that total interval length varies not only by pathway but also by year of admission.

### Contextualising individual patient experience

The data was consolidated by bringing together themes that represent participants’ journeys through the following themes: *(1) Practitioners’ Missed Opportunities for Diagnosis and Communication*,* (2) Symptom appraisal*,* interpretations and patient support system*,* (3) Disrupted continuity of care: Informal referral*,* financial insecurity and health coverage and (4) Travelling long distances for cancer care.* Demographic and clinical characteristics of interviewees (*N* = 46) were summarized and presented in a Supplementary Material 4: Table S1 Characteristics of interviewed lung cancer patients at the INCAN.

### Practitioners’ Missed Opportunities for Diagnosis and Communication

This study revealed that only 22% had never smoked, 7% reported living in houses made of asbestos, and 22% were exposed to biomass combustion due to the use of wooden stoves to cook or heat their homes since childhood. Despite these risks, 76% of patients had never undergone a lung health check prior to diagnosis.

“[Patient cries], we don’t have the education to take care of ourselves and that there is nothing that teaches us in basic education about how to prevent lung cancer. It’s like administration, you do a job and you learn but they don’t teach you how to manage resources…” - *Patient No. 45*

In this study the median number of doctors visited before the INCAN was three. At first medical encounter, 52% were misdiagnosed, commonly with infectious disease, allergies, asthma, musculoskeletal conditions, pulmonary oedema, chronic pulmonary disease, cardiomyopathy, gastritis, urinary infection, or COVID-19. In fact, 48% of the patients were prescribed an anti-inflammatory or antibiotic during their first consultation. Regardless of the symptoms or the potential diagnosis, the first doctor’s visited by the patients decided to get X-rays in 41% of the cases, biopsy in 4% of the cases, tomography 30%, sputum cytology in 2% or other (13%). Nonetheless, 22% had no laboratory or imaging follow-up. Overall, 15% of patients were eventually referred to another doctor.

*“I was at the Mexican Social Security Institution (IMSS)*, *they gave me an appointment with the oncologist and then he told me that he was going to admit me to do studies, but apparently you have to be almost dying for them to tend to one. The doctor told me that I was fine and that there were others that were worse, and they only gave me painkillers. I arrived at a very advanced stage and I think that perhaps I could have arrived earlier*, *although perhaps not. The orthopaedist did not realize that I already had bone metastases…”- Patient No. 26*

Even after diagnosis, patients reported receiving insufficient information from oncologists regarding their prognosis. Many felt their cancer diagnosis was communicated in a limited or incomplete way, leaving them uncertain about their condition and next steps.

### Symptom appraisal, interpretations and patient support system

In this study, 89% of patients noticed symptoms first (commonly cough (37%), dyspnoea (15%), back pain (7%), weight loss (4%), chest pain (4%), other symptoms (22%)). In contrast, 9% were diagnosed through a medical procedure and 2% via routine visits.

*“They were going to operate on me for a cataract but they didn’t want to because they wanted the result of the neurologist due to my diagnosis of schizophrenia and they found a tumour... I had complications, severe pain in my stomach and back*. *They gave me paracetamol. Everything stopped because emotionally it affected me a lot... But when all the pain started, then we looked for help and went to the hospital and then they said that it was cancer." - Patient No. 5 *

Across the continuum patients reported weight loss, cough, fatigue, dyspnoea, loss of appetite, chest/shoulder pain, back pain, and rarely haemoptysis. Cough was often attributed to environmental exposures (wood smoke, pesticides, coal). Weight loss was linked to life events (e.g., bereavement, poverty). Dyspnoea and fatigue were interpreted as a “normal part of aging.”

Patients often interpreted symptoms through personal or cultural frameworks. For instance, cough is often viewed as a “normal” lifelong condition or dyspnoea attributed to body weight or “old age.” Also, 30% of patients believed their symptoms were “momentary” and would resolve with self-medication or traditional remedies. Additionally, patients delayed seeking care when symptoms were not painful. Pain acted as a threshold symptom: only when it became apparent did patients feel justified in accessing medical care, to avoid “burdening” family members.

*“I thought it was because I was overweight*,* but I I never imagined that I had any lung problems. In fact, I thought that the symptoms I had were related to the smoke from making the tamale (zacahuil), because I used a brick oven. We have been making zacahuil for 23 years. We did not know that the smoke caused that. Just like smoking causes cancer." - Patient No. 17*

**Fig. 3:**
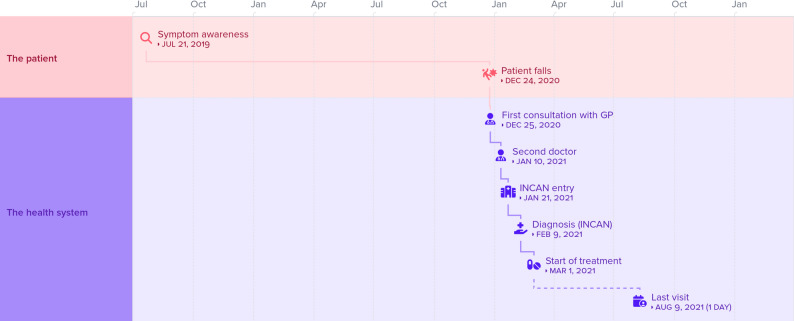
Representation of the patient journey (Patient No. 3)

Following the sicker quicker paradox, patients with more severe or acute symptoms (e.g., aphasia, seizures, vision loss) sought care more promptly. For instance, patient No. 3 in Figure  3 described being aware of some symptoms but not necessarily linking them with a disease nor believing they were serious. Hence, not until the patient falls, is it clear that something is off. The patient then quickly navigates the system and enters the INCAN about a month later. Patient 4 also portrays a similar situation.

*“I had involuntary movements in my left arm and left leg*,* I went up the stairs and fell and hit my head*. *When they did a CT scan, they diagnosed me with brain metastases of unknown origin and from there they realized it’s from the lungs. At first I thought it could be Parkinson’s and later we saw that it wasn’t…"- Patient No. 4*

Certain symptoms, particularly chronic cough, dyspnoea, and systemic changes such as weight loss — were most likely to prompt patients or their families to seek care. Patients reported that when cough became persistent and non-contagious, it raised suspicions and justified medical consultation. When patients were younger, incapacitating dyspnoea was especially salient, often leading to quicker help-seeking. Family members and friends frequently noticed weight loss, which acted as an external trigger to seek care.

Most patients first discussed their symptoms with family members (spouses, children, siblings, parents) rather than with a doctor. The average time from symptom onset to discussing concerns with family was 18 days (median: 7 days). Family persistence often played a critical role in breaking pauses in help-seeking and facilitating access to care.

*“First I told my children to leave me*,* yes*,* not to do anything to me. That I stay like this. But they insisted that they cure me. Well then whatever.” - Patient No. 41*.

**Fig. 4:**
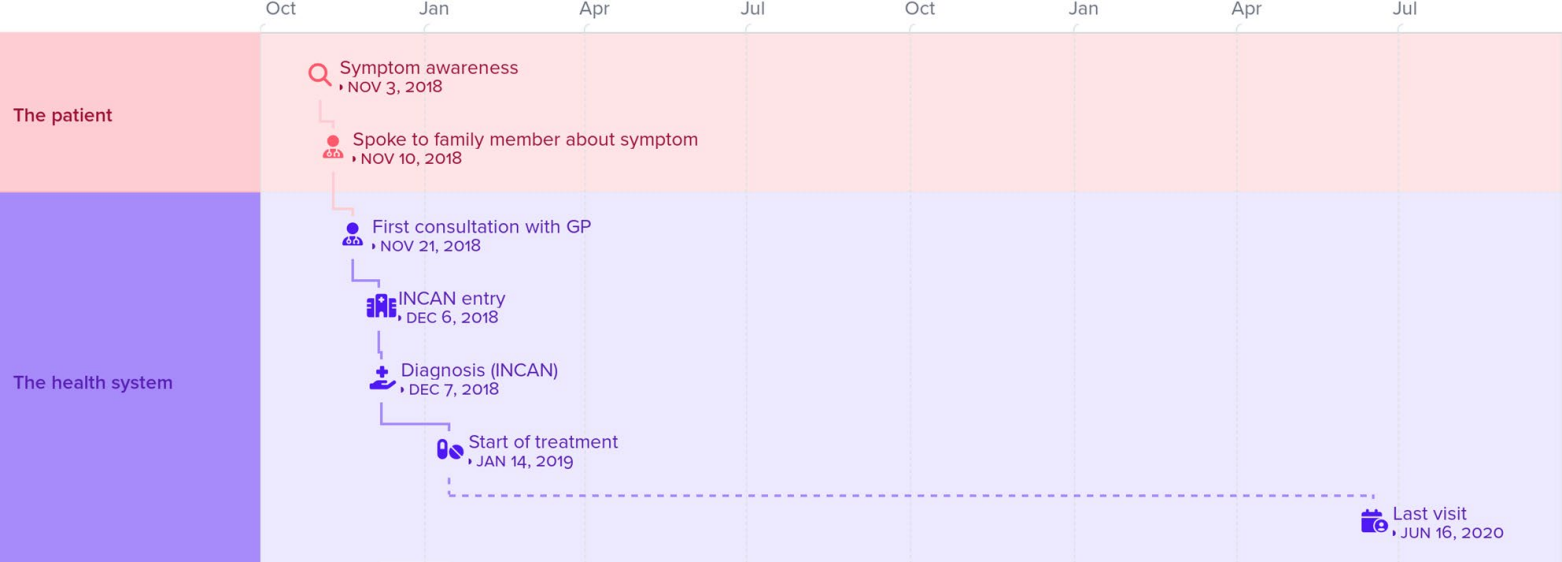
Representation of the patient journey (Patient No. 25)

Also, knowledge of family history of cancer sometimes motivated both patients and relatives to act more quickly (See Figure 4, patient No. 25).

A sense of duty or guilt also played a role. Even if the patient perceived enough reason to discuss their symptoms with a doctor, they sometimes would prefer not to be a burden to the family or to avoid the family members spending money on them. Additionally, in 20% of the patients’ fear was an important factor that interfered with the patients’ desire to seek medical attention. For instance, patients reported being afraid of being revised by a medical doctor and therefore withheld from seeking care. Moreover, 24% of patients described not being able to seek care the first time because they couldn’t get off work. In addition, 9% referred to not being able to seek care due to having to care for someone (child, elderly or other). In the interviews, patients were worried about losing their job or losing money over seeking care.

### Disrupted continuity of care: Informal referral, financial insecurity and health coverage

Results from the interviews indicate that lung cancer patients frequently used private and a mix of private and public services. Most patients (80%) reported having no health insurance, while 17% were insured through the Mexican Social Security Institute (IMSS) (for private-sector employees) and 2% through the Institute for Social Security and Services for State Workers (ISSSTE). Nonetheless, some patients reported disenrolling from IMSS to seek care at INCAN. While this decision enabled them to access lung cancer treatment, it left them without coverage for other conditions (e.g., COVID-19, diabetes, surgical care). Patients acknowledged this as a difficult trade-off but viewed it as necessary to continue cancer treatment.

Participants who lost IMSS coverage, often due to unemployment or illness, described being forced to discontinue treatment. They reported navigating multiple systems (e.g., INSBAI Institute of Health for Well-Being, private clinics), which increased administrative and clinical burdens and caused significant delays. Other participants reported exhausting private insurance budgets, which forced them to shift to the public system. In the absence of a clear referral pathway, these patients often incurred catastrophic expenses and personally managed the transition between providers, leading to delays. Upon this change in the healthcare landscape, patients frequently expressed uncertainty about their eligibility for specific treatments, leading to feelings of despair and disorientation within the health system.

Nearly half of the participants expressed concern about covering treatment costs, while 28% specifically worried that lack of insurance would exclude them from diagnosis or treatment altogether. Costs accumulated quickly due to consultations, diagnostic tests, treatments, and supportive care. Patients described the financial strain forcing them to prioritize basic needs (food, housing, and transportation) over medical expenses. This financial burden resulted in treatment delays, interruptions, poor adherence to medications, and compromised follow-up care.

*“You spend what you don’t have in an attempt to survive. It’s draining*,* both physically and financially…” - Patient No. 26*.

Patients described how informal referrals between physicians across institutions often shaped access to cancer care. For example, oncologists working both at IMSS and INCAN facilitated access by personally referring patients. However, when such connections were absent, patients had to navigate the system on their own, leading to delays.

*“The truth is that we waited a long time at the IMSS*, *Dr. XXX recommended that we come here due toour economic situation. This journey was difficult because the hospital is far away. We tried to enter here initially*,* but they didn’t accept us. We sent an email*,* and they didn’t accept us either. They only accepted us when we mentioned that Dr. XXX recommended us…” - Patient No. 11*.

According to the interviews, a median of three medical encounters took place before being admitted to the INCAN. Across cases, patients consistently identified challenges such as repeatedly recounting medical histories due to lack of file portability, duplicate diagnostic testing, searching for second and third opinions, long travel distances, fear of disease progression and COVID-19.

### Travelling long distances for cancer care

Some reported that, even when infrastructure and medical staff were available in nearby facilities, access was denied if they lacked affiliation with the corresponding insurance scheme or could not pay for private services. As a result, patients often had to travel long distances—sometimes by plane or several hours by bus— to institutions where they could be seen, despite having hospitals much closer to home. According to the interviews, it took patients approximately 1.5 h to reach the INCAN. In some cases, diagnosis and treatment were still not provided upon arrival. Patients described their healthcare “landscape” as rigid and exclusionary, leading to frustration, anger, and anxiety over delayed access to care.

*“They never gave me a diagnosis. They took fluid from me many times and the diagnosis did not come out and it hurt a lot…” - Patient No. 13*.

Patients consistently reported long waits for diagnosis, sometimes receiving an initial misdiagnosis or needing to pay out-of-pocket for private consultations to reach a confirmed result. Many described travelling to urban centres to access specialized services, as diagnostic infrastructure was unavailable in their local facilities. Several patients shared that when they did reach facilities, equipment was often broken and they were not informed when it would be functional again, leaving them without follow-up and contributing to further delays. Upon arrival at INCAN, patients sometimes faced the requirement to repeat biopsies because prior samples were inadequate or not performed according to institutional standards.

*“I come to the state capital (Pachuca to H. Gral) and the oncologists told me that they didn’t have thein frastructure to do the studies or the therapies and so they sent me here (INCAN)…”- Patient No. 17*.

### Linking measured delays with lived experience and pathway

Table 2 presents the joint-display of electronic record data and interview data, joining information regarding the diagnostic pathway (symptoms, screening or clinical finding), the combination of actors/institutions encountered throughout their journey, the identified health system user (private healthcare, public healthcare users and mixed users), and the time intervals for each patient (health system, appraisal and total interval) if registered.

**Table 2:** Joint display for data collected from interviews and electronic health records

Patient No.	Diagnostic pathway**	Presentation*	First Symptom*	First diagnosis*	No. of Actors encountered*	Actors visited in order*	User type**	Hours to INCAN*	Transportation used*	Total interval	System interval*	Patient interval*
1	Diagnosis external & INCAN	Health Check-up	NA	Malignant tumour	2	CB	Public					69
2	Diagnosis external & INCAN	Finding	NA	Other, not cancer	2	HD	Private	1.26	car,taxi	215	143	72
3	INCAN Diagnosis only	hallazgo durante otro procedimiento o diagnóstico	NA	Suspected cancer	2	DD	Private	1.5	car	589		
4	INCAN Diagnosis only	hallazgo durante otro procedimiento o diagnóstico	NA	Malignant tumour	3	DHH	Private	1.34	car	85		
5	INCAN Diagnosis only	Finding	NA	Suspected cancer	4	CCBG	Public	0.67	public van, taxi	89		
6	INCAN Diagnosis only	Symptomatic	Dyspnoea	Suspected cancer	3	EID	Mixed	0.84	car		36	52
7	Undetermined	Symptomatic	Dyspnoea	Other, not cancer	2	DD	Private	1	car		149	55
8	Undetermined	Symptomatic	Other	Other, not cancer	5	DDDDD	Private	3.5	public van			69
9	Undetermined	Symptomatic	Other	Suspected cancer	3	FJD	Mixed	0.34	car, uber			
10	Undetermined	Symptomatic	Chest pain	Other, not cancer	5	GDDDD	Mixed	4	coach, bus			
11	INCAN Diagnosis only	Symptomatic	Cough	Suspected cancer	3	HAD	Mixed	3	car, uber		37	43
12	INCAN Diagnosis only	Symptomatic	Cough	Suspected cancer	2	HH	Private	0.5	car		202	144
13	Undetermined	Symptomatic	Cough	Other, not cancer	5	DDCDD	Mixed	1.5	car		350	56
14	External diagnosis only	Symptomatic	Cough	Other, not cancer	7	HHHHHHH	Private	2	car			
15	Undetermined	Symptomatic	Undefined	Other, not cancer	3	HHH	Private	3			281	43
16	External diagnosis only	Symptomatic	Weight-loss	Other, not cancer	4	GGDC	Mixed				143	72
17	Undetermined	Symptomatic	Dyspnoea	Other, not cancer	3	DHH	Private	7	bus			
18	External diagnosis only	Symptomatic	Other	Other, not cancer	13	HHAAHHHHHHEDD	Mixed	2	car			
19	Undetermined	Symptomatic	Other	Other, not cancer	2	ED	Private	0.5	car		83	31
20	Undetermined	Symptomatic	Cough	Other, not cancer	4	EDID	Mixed		metro, public van		161	66
21	Undetermined	Symptomatic	Cough	Other, not cancer	3	DDB	Mixed	4	bus, taxi		97	70
22	Undetermined	Symptomatic	Other	Other, not cancer	5	EDDDB	Mixed	2.5	metro, public van, bus		30	240
23	Undetermined	Symptomatic	Other	Other, not cancer	2	DD	Private					
24	Undetermined	Symptomatic	Cough	Suspected cancer	2	FB	Mixed	0.26	car			
25	INCAN Diagnosis only	Symptomatic	Dyspnoea	Suspected cancer	1	D	Private			60		
26	Diagnosis external & INCAN	Symptomatic	Cough	Suspected cancer	6	EIAIDD	Mixed	2	car	378	4	374
27	Diagnosis external & INCAN	Symptomatic	Chest pain	Malignant tumour	3	EDB	Mixed			393	319	74
28	INCAN Diagnosis only	Symptomatic	Other	Suspected cancer	1	D	Private			446		
29	INCAN Diagnosis only	Symptomatic	Cough	Suspected cancer	2	EA	Mixed			740		
30	Diagnosis external & INCAN	Symptomatic	Cough	Suspected cancer	3	DIB	Mixed			88	36	52
31	Diagnosis external & INCAN	Symptomatic	Cough	Other, not cancer	4	EADI	Mixed			204	149	55
32	INCAN Diagnosis only	Symptomatic	Cough	Other, not cancer	2	DDD	Private					
33	INCAN Diagnosis only	Symptomatic	Cough	Other, not cancer	3	DDD	Private			254		
34	Diagnosis external & INCAN	Symptomatic	Cough	Suspected cancer	2	FD	Private			80	37	43
35	Diagnosis external & INCAN	Symptomatic	Dyspnoea	Malignant tumour	4	EDCB	Mixed			346	202	144
36	Diagnosis external & INCAN	Symptomatic	Cough	Other, not cancer	1	D	Private			406	350	56
37	Diagnosis external & INCAN	Symptomatic	Weight-loss	Suspected cancer	3	DDD	Private	1	metro, taxi	324	281	43
38	INCAN Diagnosis only	Symptomatic	Other	Other, not cancer	3	DDC	Mixed	3	taxi	369		
39	Diagnosis external & INCAN	Symptomatic	Back pain	Other, not cancer	3	HHC	Mixed	1	taxi	114	83	31
40	Diagnosis external & INCAN	Symptomatic	Cough	Suspected cancer	3	DDD	Private	6.67	car, plane, taxi	227	161	66
41	Diagnosis external & INCAN	Symptomatic	Dyspnoea	Other, not cancer	4	DDDD	Private	3	public van, taxi	167	97	70
42	Diagnosis external & INCAN	Symptomatic	Back pain	Suspected cancer	3	DDD	Private	2.5	car	270	30	240
43	INCAN Diagnosis only	Symptomatic	Back pain	Other, not cancer	4	DEDD	Private	1.5	metro,public van, bus			
44	Diagnosis external & INCAN	Symptomatic	Other	Malignant tumour	1	B	Public	1.34	taxi	59	37	22
45	INCAN Diagnosis only	Symptomatic	Other	Suspected cancer	4	HHDH	Private	2	car	38		
46	Diagnosis external & INCAN	Symptomatic	Cough	Other, not cancer	2	DD	Private	1.5	car,metro			29
				***General Median***	**3**			**1.5**		***227***	***143***	***61***

1-At the top: *Marks the data collected from interviews. **Marks new data arising from the analysis. The rest are data collected from health records. 2-The different institutions were coded: Mexican Social Security Institute (IMSS) (second level of care) = A; National Health Institute = B; Hospital MOH (third level of care) = C; Private hospital = D; Private pharmacy doctor = E; Private laboratory = F; First level of care MOH = G; Private practitioner = H; Hospital IMSS (third level of care) = I; Social Insurance Institutions for Government Workers (ISSSTE) Hospital (third level of care) = J

## Discussion

In the literature, timeliness and access to lung cancer care are influenced by multiple factors, including cancer subtype, age, race, education, socioeconomic position, rurality, employment, insurance status, type of radiotherapy (curative vs. palliative), referral route, number of diagnostic tests prior to diagnosis, number of hospitals and specialists involved, lack of multidisciplinary team assessment, comorbidities, and atypical or absent symptoms at presentation [3, 16, 31–33, 37–41].

Like other countries, the lung cancer journey in Mexico is marked by moments of introspection and self-awareness [42]. Upon receiving a diagnosis of lung cancer, the initial response of the majority of patients is disbelief, often expressing sentiments like “this can’t be true” or “it must be a mistake“ [42]. During this period of self-reflection, patients actively explore possible factors that might have contributed to their illness, seeking to understand any personal behaviours or habits that could have played a role in the development of their condition [42]. Fear of doctors and fear of the truth are present [43] as well as a sense of guilt that shadows the patient’s urge to seek care [42]. Nevertheless, evidence suggests that as time progresses, most patients gradually come to terms with the reality of their diagnosis and begin to comply with the treatment plan recommended by their healthcare provider [42]. Nevertheless, our findings reveal that throughout this difficult personal journey, in addition to many patients experiencing barriers in their pathway to diagnosis and treatment in Mexico due to the malfunctioning referral system between institutions [16–18, 21], infrastructure centralisation [44] and health system fragmentation [18].

Our study shows that most patients admitted to the INCAN reside in Mexico City and are referred from the private sector in both the broader and interviewed samples. Therefore, it appears that we may be dealing with a population whose characteristics differ significantly from those of the general population. Suggesting that these patients are the best fit to navigate a fragmented health system. Potentially, these patients find the private sector to be more accessible considering factors such as distance, economic cost, and social cost. In breast cancer, literature from Mexico suggests that choosing the private care pathway renders the patients with fewer delays and a significant addition to survival time [45]. Thus, the identification of journey typologies in this study provides valuable insights into the different healthcare-seeking patterns and preferences of patients, as well as the perception of patients with regards to the public health system. Nonetheless, even with this “privileged” sample these patients will still experience barriers in accessing treatment [16, 20]. All of these structural gaps result in health inequalities, treatment delays, treatment interruptions, and increased out-of-pocket costs [15]—patterns that align with global evidence on the financial toxicity associated with cancer care [46, 47].

Capturing patients’ strategic disenrollment from IMSS to access INCAN illustrates how individuals attempt to navigate these barriers, but at the cost of losing coverage for other essential health services. This reflects a perverse incentive within the system, where fragmented insurance forces patients into difficult trade-offs that ultimately undermine comprehensive, continuous care. Moreover, the lack of formal referral pathways between healthcare providers and the lack of urgent referral pathways [48, 49] contribute to further longer intervals. In contrast, informal referrals from informal physician networks streamline care but benefit only a few, reinforcing inequalities by favouring those with personal connections and leaving others behind. This reflects broader patterns of “healthcare brokerage” observed in fragmented systems, where access depends not solely on clinical need, but also on social capital [50].

Unfortunately, according to our study, practitioners missed critical opportunities for early diagnosis. Despite clear exposure to well-established risk factors, most patients had never received a preventive lung check-up. This highlights the lack of systematic screening for high-risk groups. Practitioners and hospitals could implement risk assessment tools to promote timely access to suspected lung cancer cases, especially for patients with risk factors that are experiencing severe, unusual, or changing symptoms that may indicate underlying health conditions [51]. However, this is harder to achieve when the system doesn’t share electronic health records and the system is fragmented [49]. Patients might be sharing their symptoms with physicians from different institutions and the private sector, but they are all unaware of this. Thus, our study highlights that seamless data linkage between different healthcare institutions (public or private) throughout a cancer pathway is increasingly necessary [17]. This will allow the patient’s information to be accessed without interrupting the cancer care pathway [52] and will avoid duplicative clinical assessments. It is now imperative to have unique identifiers and electronic records to not only allow research on cancer pathways and examine cancer intervals across care and facilities [52], but also to allow the patient to navigate the system without losing any time or data.

Additionally, the high rate of initial misdiagnosis in our sample reflects a low capacity among frontline providers to recognize early signs of lung cancer. Symptoms were frequently misattributed to more common conditions, resulting in inappropriate treatments such as antibiotics or anti-inflammatory medication. This mirrors existing literature on how non-specific symptoms often delay cancer recognition in primary care [5].Our study shows the pathway to treatment is not standardised. Although in low- and middle-income countries X-ray remains the chosen method for diagnosis, with sensitivity ranging 77%–80%, for the diagnosis of symptomatic lung cancer [53], some patients were directly sent to a tomography scan or biopsy. Some patients received no follow-up investigations at all, exposing gaps in clinical suspicion, continuity of care, and the broader diagnostic pathway, all of which contribute to late-stage diagnosis. This seems to be consistent with previous studies that interviewed oncologists, patients, and family members of patients with lung cancer and identified multiple barriers for early diagnosis and treatment, such as medical time constraints for patient examination, misdiagnosis, and lack of training [16]. In consequence, more capacity needs to be built in primary and secondary care.

Accurate diagnosis, staging, and treatment often require travel to urban centres with appropriate infrastructure [20, 21, 44]. While some patients were correctly diagnosed outside of INCAN our results show that INCAN continues to shoulder a disproportionate burden of diagnosis. Patients’ reports of long travel distances and repeated biopsies reflect broader systemic issues, including the limited availability and uneven distribution of diagnostic resources. Patients have previously complained about the reduced workforce [16], insufficient infrastructure [16, 44], but according to other studies, it could also be because diagnostic tests performed at other hospitals are often of inadequate quality, requiring repetition [32]. As a result, duplication not only delays care but also indicates the urgent need to strengthen diagnostic capabilities outside INCAN. A more equitable system would ensure that regional hospitals can conduct high-quality biopsies and imaging, eliminating the need for repeated testing. More research is needed to explore the institutional barriers between healthcare levels, including provider perceptions of diagnostic reliability at other facilities. Understanding whether mistrust, technical limitations, or other beliefs influence INCAN’s reliance on repeat diagnostics will be critical. Such inefficiencies come at a financial cost that the health system cannot afford [20].

Our findings suggest that both symptom signatures [5] and social context strongly influence this process symptom appraisal. Patients often required validation from family members before acting on symptoms like persistent cough or dyspnoea, while those without such support tended to delay care. This adds to previous research describing individual-level barriers such as symptom interpretation, fear of diagnosis, and disruptions to personal life, and the heavy burden of caregiving from family members [16]. Hence, awareness campaigns [54] should adopt a family-centred approach, targeting not only high-risk individuals but also their caregivers [55]. These should focus on risk factors (other than smoking) and challenge normalization narratives—such as attributing cough to wood smoke or fatigue to aging—and leverage personal experiences, like family history of cancer, to prompt earlier help-seeking. Explaining the procedure for clinical examination during these campaigns might also help mitigate fear. In parallel, practitioners should learn about the patients’ cultural backgrounds and misconceptions surrounding symptoms in lung cancer [27, 41], making it easier for them to interpret the situation at the time of clinical presentation.

Overall, a larger number of medical encounters are regarded as a less positive experience in cancer care due to their association with prolonged diagnosis and treatment [56, 57]. Through Table 2 we see lung cancer patients in Mexico often undergo numerous consultations before being referred to a hospital. However, the median number of doctors visited identified through interviews should not be generalisable.

The findings highlight several implications for practice. The health system should be better integrated through standardized referral and diagnostic protocols and coordinated pathways across providers to reduce delays and missed follow-up. Regional cancer centres should be strengthened through investment in diagnostic infrastructure, functional equipment, reliable maintenance, and quality assurance in biopsy and pathology services to ensure timely treatment. Financial protection mechanisms should also be expanded so that access to life-saving cancer care does not depend on employment status, including the inclusion of lung cancer in the catastrophic expenditure fund. In addition, primary and secondary care should be reinforced through targeted surveillance of high-risk populations (such as smokers and individuals exposed to asbestos or biomass smoke), improved provider capacity to recognize lung cancer warning signs, and patient awareness initiatives. Finally, communication practices should be improved to ensure patients receive clear and comprehensive information about their diagnosis, treatment options, and prognosis.

Qualitative data obtained from this study may not have yielded generalisable insights into all the experiences of all lung cancer patients across Mexico. Future efforts to collect larger sets of data is encouraged to evaluate other regions and patients being admitted to other hospitals besides INCAN.

Validating the structured interview’s bias and protocol adherence could bolster the trustworthiness of the findings. Additionally, the original plan was to conduct interviews as soon as possible after diagnosis; however, in practice this was not always feasible due to missed follow-up appointments, pain, or the constraints imposed by COVID-19 [58]. Extending the recruitment window from 6 months to 12 months introduced the possibility of recall bias, as patients were asked to reflect on experiences that occurred several months earlier. This may have affected the precision of their accounts of timelines, decisions, and interactions. Nevertheless, the themes developed from the data reflected consistent patterns across patients recruited both before (groups A-D) and during the pandemic (Group E). These findings therefore represent the broader experiences of patients along the lung cancer care journey and suggest stability in the core challenges identified, regardless of the timing of the interview.

## Conclusions

This study examined the lung cancer care journey through patient narratives and complemented these insights with the analysis of time intervals and care pathways. Results highlight the importance of stratifying research by patients’ diagnostic and treatment pathways to identify outcome inequalities and potential inefficiencies in the health system. Similarly, categorizing them into private, public, or mixed healthcare users allows us to further understand the health brokerage phenomenon. To address these inequities, we recommend major efforts to integrate the health system, strengthen regional cancer centres, implement financial protection mechanisms, enhance targeted surveillance among high-risk populations, expand provider training, and intensify awareness campaigns and patient education. These actions are urgent and essential not only to reduce delays across the continuum of care but also to improve the quality of life and clinical outcomes for lung cancer patients in Mexico.

## Supplementary Information

Below is the link to the electronic supplementary material.

Supplementary Material 1: Semi-structured interview guide for lung cancer patient journey

Supplementary Material 2: Codebook for qualitative analysis of lung cancer patient journey

Supplementary Material 3: COREQ Checklist

Supplementary Material 4: Characteristics of interviewed lung cancer patients at the INCAN (N=46)

## Data Availability

Available data is presented in this paper in Table 2. Due to the sensitive nature of the data, necessary precautions were taken to protect the participants’ identities. Electronic health records cannot be shared. For further questions related to data, please contact the corresponding author (a.peasey@ucl.ac.uk).

## Abbreviations

CONACYT: Consejo Nacional de Humanidades, Ciencias y Tecnologías—National Council of Humanities, Sciences and Technologies (Mexico)
COREQ: Consolidated Criteria for Reporting Qualitative Research
ECOG: ECOG Performance Status – Eastern Cooperative Oncology Group Performance Status
IARC: International Agency for Research on Cancer
IMSS: Instituto Mexicano del Seguro Social – Mexican Social Security Institute
INCAN: Instituto Nacional de Cancerología – National Cancer Institute (Mexico)
INSABI: Instituto de Salud para el Bienestar – Institute of Health for Well-being
ISSSTE: Instituto de Seguridad y Servicios Sociales de los Trabajadores del Estado—Institute for Social Security and Services for State Workers
NRC: National Research Council Canada
UKRI: UK Research and Innovation
