# Supplementary material for: Lung cancer care pathways and journeys: insights from patients at the National Cancer Institute in Mexico
Source: BMC Glob Public Health. 2026 May 27;4:50. doi: 10.1186/s44263-026-00278-7 (PMC13214188; doi:10.1186/s44263-026-00278-7)
Supplement: Supplementary file 2 — Supplementary Material 2: Codebook for qualitative analysis of lung cancer patient journey [file 44263_2026_278_MOESM2_ESM.pdf]

Patient trajectories and delays to lung cancer care

by Elysse Bautista Gonzalez

File number

____
(Identification number)

Level of conciousness (ECOG)

◯ 0
◯ 1
◯ 2
◯ 3
◯ 4

Date of birth

____
(dd/mm/aa)

Phone number or mobile

____
(Home or mobile)

Sex

◯ Woman
◯ Men

Was spanish your first language?

◯ Yes
◯ No

If not which is it?

____
(Dialect or other language)

Do you know how to read and write?

◯ Yes
◯ No

Education level

◯ Incomplete elementary
◯ Complete elementary
◯ Incomplete middle-school
◯ Complete middle-school
◯ Incomplete high-school
◯ Complete high-school
◯ Incomplete University
◯ Complete University
◯ MSC or PhD
◯ No formal education

State of residency

- ◯ Aguascalientes
- ◯ Baja California
- ◯ Baja California Sur
- ◯ Campeche
- ◯ Chiapas
- ◯ Chihuahua
- ◯ Ciudad de México
- ◯ Coahuila
- ◯ Colima
- ◯ Durango
- ◯ Estado de México
- ◯ Guanajuato
- ◯ Guerrero
- ◯ Hidalgo
- ◯ Jalisco
- ◯ Michoacán
- ◯ Morelos
- ◯ Nayarit
- ◯ Nuevo León
- ◯ Oaxaca
- ◯ Puebla
- ◯ Querétaro
- ◯ Quintana Roo
- ◯ San Luis Potosí
- ◯ Sinaloa
- ◯ Sonora
- ◯ Tabasco
- ◯ Tamaulipas
- ◯ Tlaxcala
- ◯ Veracruz
- ◯ Yucatán
- ◯ Zacatecas

Have you any of the following health insurance?

- ☐ INSABI
- ☐ ISSSTE
- ☐ IMSS
- ☐ PEMEX
- ☐ SEDENA
- ☐ SEMAR
- ☐ Ninguno
- ☐ Other*

Marital status

- ◯ Single
- ◯ Married
- ◯ Divorced
- ◯ Widowed

Laboral status

- ◯ Employed
- ◯ Unemployex
- ◯ Doesnt work
- ◯ Retired
- ◯ Unemployed due to COVID

Monthly income

____
MXN

Risk factors for lung cancer

	Yes	No
Smoking	◯	◯
Passive smoker	◯	◯
Asbestos	◯	◯
Biomass	◯	◯

Smoking index ______________________

Patient trajectories and barriers to diagnosis and treatment

How did you realise you were sick?

- ◯ I developed symptpms
- ◯ Routine health check
- ◯ Lung cancer screening
- ◯ Finding during another event

What was the first symptom you noticed?

- ◯ hemoptysis
- ◯ chest pain
- ◯ shoulder pain
- ◯ dyspnea
- ◯ cough
- ◯ loss of appetite
- ◯ weight loss
- ◯ Other (s)

*What other first symptom?

____

Additional symptoms

- ☐ chest pain
- ☐ Shoulder pain
- ☐ back pain
- ☐ cough
- ☐ hemoptisis
- ☐ fatigue
- ☐ weightloss
- ☐ loss of appetite
- ☐ dyspnea (shortness of breath)
- ☐ other pulmonary symptoms
- ☐ other extra-pulmonary symptoms

Other*

____

Which of the symptoms mentioned above were you most concerned about?

- ◯ chest pain
- ◯ Shoulder pain
- ◯ back pain
- ◯ cough
- ◯ hemoptisis
- ◯ fatigue
- ◯ weightloss
- ◯ loss of appetite
- ◯ dyspnea (shortness of breath)
- ◯ other pulmonary symptoms
- other extra-pulmonary symptoms

When did you first identify the first symptom?

____
(dd-mm-aaaa)

Tell me, what did you think of these symptoms / ailments / discomforts?

____

Who is the person you first talked to about your illness?	◯ Father<br>◯ Mother<br>◯ Child (son/daughter)<br>◯ Spouse<br>◯ Friend<br>◯ Colleague<br>◯ Sibling<br>◯ Other<br>◯ Physician
How long was it between the first time you noticed the symptoms and the time to talk about them (in days)?	__________________<br>(days)
When you first noticed your symptom, how serious did you think it was?	◯ nothing serious<br>◯ somewhat serious<br>◯ moderately serious<br>◯ serious<br>◯ very serious
How concerned were you about this symptom back then?	◯ not worried at all<br>◯ a little worried<br>◯ somewhat worried<br>◯ very worried<br>◯ does not respond
When you first noticed this symptom, did you think it might be related to cancer?	◯ Yes<br>◯ No
How many hospitals / doctors did you visit before arriving at INCAN?	__________________
List ALL the actors you have met before being admitted to INCAN (for the same illness)	☐ IMSS UMF (A)<br>☐ National Institute (B)<br>☐ another public hospital of second or third level of SSA (C)<br>☐ another private hospital (D)<br>☐ private pharmacy office (E)<br>☐ private laboratory (F)<br>☐ First Level Clinic (SSA) (G)<br>☐ IMSS (second or third level) (I)<br>☐ ISSSTE (second or third level) (J)<br>☐ Other * (K)
What other?	__________________
Please order the actors you selected in a sequential order. (Actors can be repeated i.e. A-> E-> E-> B)	__________________
When did you first see the first doctor?	__________________<br>(dd-mm-aaaa)

With the first healthcare professional you met: What did he / she say about your lungs?	◯ benign tumor<br>◯ suspicious tumor<br>◯ malignant tumor<br>◯ other *
*What other?	__________________________
What studies did the first doctor you spoke to ask for to study your first symptom or finding?	☐ biopsy<br>☐ tomography<br>☐ X-rays<br>☐ sputum cytology<br>☐ None of the above<br>☐ The patient was referred to another doctor.<br>☐ No study
Were you prescribed an anti-inflammatory or antibiotic during your first visit?	◯ Yes<br>◯ No
Date of visit and distance from the house to the actors mentioned above	__________________________<br>(i.e.: E, 01/01/20, 15 minutes and D if diagnosed)
What do you think of this path (coming and going) from doctor to doctor?	__________________________
What made you decide to seek medical attention?	☐ that the symptoms mentioned above could come back<br>☐ that the symptoms mentioned above could worsen<br>☐ that the symptoms mentioned above could interfere with your usual activities: family<br>☐ counseling or social network<br>☐ anything else*
*What else?	__________________________
How much do you think it was difficult for you to go to the doctor that first time?	◯ Very hard<br>◯ Hard<br>◯ Somewhat hard<br>◯ A little hard<br>◯ Not hard at all

Why has it been hard to reach medical care?

	Si	No
Due to laziness	◯	◯
I thought the problem was going to go away	◯	◯
Because I didn't want to stop working	◯	◯
I did not have money to cover the expenses generated by my illness	◯	◯
For not knowing the open / available medical services due to COVID-19	◯	◯
I thought I didn't have any health insurance to cover me	◯	◯
Because I have to take care of a family member (children, the elderly or others)	◯	◯
Because I was afraid of knowing my diagnosis	◯	◯
Because I didn't want to be examined	◯	◯
Fear of seeking care for COVID-19	◯	◯
For another reason * (describe)	◯	◯

Other*: ________________________________

Because of the disease, have you had to stop doing some activities?
- ◯ Yes
- ◯ No

What kind of activities?
- ☐ job
- ☐ cleaning household
- ☐ favorite activity
- ☐ family care
- ☐ other *

When did you first arrive at INCAN? (date) ________________________________

Why did you come here (to INCAN)?
- ◯ on my own initiative
- ◯ through the advice of a friend or relative
- ◯ a doctor / hospital sent me

What institution sent you here (to INCAN)?	◯ primary health care health<br>◯ clinic<br>◯ general hospital<br>◯ national institute<br>◯ private medical services<br>◯ pharmacy<br>◯ IMSS<br>◯ ISSSTE<br>◯ others *
*Others	__________________________________
Where were you diagnosed PRIOR to joining INCAN?	◯ Yes<br>◯ No
Date of diagnosis (external institution)	__________________________________
Was the patient treated by the external institution?	◯ Yes<br>◯ No
Treatment start date (external institution)	__________________________________
Type of treatment granted (external institution)	☐ Chemo<br>☐ Surgery<br>☐ TKI<br>☐ Immunotherapy<br>☐ Radiotherapy
How difficult do you think the process was to receive him at INCAN?	◯ Very difficult<br>◯ Difficult<br>◯ Somewwhat difficult<br>◯ A little difficult<br>◯ Not difficult
Why do you think it was (LEVEL) difficult or took so long to get to INCAN?	____

Why was it difficult to reach INCAN?

	Yes	No
That I was afraid	○	○
I did not have the diagnosis	○	○
That an incorrect diagnosis was initially made	○	○
He had no money to pay for the medical consultation or diagnostic studies.	○	○
I did not have the information about what services I could access	○	○
The programming /scheduling of the dates	○	○
That I couldn't leave my job to go get care	○	○
That you had to take care of a family member, an elderly or sick person	○	○
That he was afraid of attending the hospital because of COVID19	○	○
Affectation of services by COVID19	○	○
Anything else*	○	○

*Other ______________________________

How many hours does it take to get to INCAN? ______________________________

What methods of transportation did you use to get to all the places you previously visited?

- ☐ Car
- ☐ Metro
- ☐ Combi
- ☐ Bus
- ☐ Taxi
- ☐ Uber
- ☐ Caminando
- ☐ ADO, Estrella Blanca, u otro bus inter-estatal

Have you had to find shelters for yourself or your family members during this navigation process?

- ○ Yes
- ○ No

So far payments for her medical consultations and treatment.

- ○ have been paid by me
- ○ have been paid by me and someone else
- ○ has been paid by someone else

Who has helped you pay for your medical treatment or doctor visits?

- ○ husband or wife
- ○ another member of the family
- ○ non-profit (NGO)
- ○ children (son,daughter)
- ○ nobody

In total, how much have you spent to pay for consultations, medications, transportation or others so far?	______________________ (MXN)
How much does it cost you to come to each consultation at INCAN? (transport)	______________________ (MXN)
Who will be available to accompany you to medical consultations?	☐ husband or wife<br>☐ son/daughter<br>☐ parents<br>☐ friends<br>☐ other family member<br>☐ coworkers<br>☐ other community groups<br>☐ nobody
Have you ever been offered a lung cancer screening test?	◯ Yes<br>◯ No
What kind of information have been given by the medical professionals who have treated you?	☐ information about my disease (lung cancer)<br>☐ information about the services provided by the hospitals or clinics for my illness<br>☐ information about the schedules for the hospitals or clinics<br>☐ information on the treatment of the disease<br>☐ information on costs of the disease<br>☐ information NOT related to my disease<br>☐ No information
If you were given information, was it useful to you?	◯ Yes<br>◯ No
What information would you have liked to have been given?	☐ Disease<br>☐ Forecast<br>☐ Treatment<br>☐ Available services<br>☐ Information on shelters<br>☐ Information on financial support<br>☐ Other*
Other*	______________________
Have you been approached by patient navigation programs to assist you during your illness?	◯ Yes<br>◯ No
Before coming to INCAN, did you trust that they were giving you the best treatment / the best care / were you in good hands?	◯ Yes<br>◯ No
Now that you are at INCAN, how do you feel?	______________________

What do you know about COVID19? ______________________________

How do you think COVID-19 has affected you?

- ☐ Access to diagnostic services
- ☐ Access to treatment services
- ☐ Fear of becoming infected
- ☐ I have not been affected by COVID-19
- ☐ Does not respond
- ☐ Other*

Other* __________________________
