# Supplementary material for: Lung cancer care pathways and journeys: insights from patients at the National Cancer Institute in Mexico
Source: BMC Glob Public Health. 2026 May 27;4:50. doi: 10.1186/s44263-026-00278-7 (PMC13214188; doi:10.1186/s44263-026-00278-7)
Supplement: Supplementary file 3 — Supplementary Material 3: COREQ Checklist [file 44263_2026_278_MOESM3_ESM.docx]

**Table S1. Socio-demographic and clinical characteristics of the population with lung cancer studied through structured interviews amongst patients (N=46)**

| **Parameters** | **Percentage/Numbers/Other** |
| --- | --- |
| Patients interviewed | 46 |
| Gender distribution | 63% men (29), 37% women (17) |
| Age | 57 years (mean), 64 years (median) |
| Metropolitan area residents | 76% (Mexico City, State of Mexico) |
| Other states | Hidalgo, Tlaxcala, Veracruz, Puebla, Guanajuato, Michoacan, Chihuahua |
| Marital status | Married - 54% (25), Single - 22% (10), Widowed - 17% (8), Divorced - 7% (3) |
| Monthly earning | Average: $3729 MXN - 171 GBP, Median: $5500 MXN - 253 GBP |
| Pathway | Patients with initial symptoms - 89% (41), Diagnosed through clinical procedures - 9% (4), Diagnosed during routine check-up - 2% (1) |
| Smoking | Current smokers - 22% (10), Former smokers - 30% (14), Never smoked - 22% (10) |
| Patients who never had their lungs checked | 76% (35) |
| Common symptoms | Weight loss, cough, fatigue, dyspnoea, loss of appetite, shoulder or chest pain, back pain, and others |
| Most common first symptom | Cough - 37% (17) |
| Other first symptoms | Dyspnoea - 15% (7), Back pain - 7% (3), Weight loss - 4% (2), Chest pain - 4% (2), Other symptoms - 22% (10) |
| Advanced disease symptoms | Dyslalia, loss of sight, seizures, depression, palpebral ptosis, headaches |
| Patients who had to stop activities/work due to cancer | 54% (25) |
| Communication about symptoms | 78% (36) with family members, 46% (21) relatives led them to seek care |
| Average time to talk to family members | 18 days |
| Patients who never suspected cancer-related | 80% (37) |
| Insurance Type | Patients with no insurance - 50% (23), Patients insured by IMSS - 17% (8), Patients insured by ISSSTE - 2% (1) |
