# Supplementary material for: Lung cancer care pathways and journeys: insights from patients at the National Cancer Institute in Mexico
Source: BMC Glob Public Health. 2026 May 27;4:50. doi: 10.1186/s44263-026-00278-7 (PMC13214188; doi:10.1186/s44263-026-00278-7)
Supplement: Supplementary file 4 — Supplementary Material 4: Characteristics of interviewed lung cancer patients at the INCAN (N=46) [file 44263_2026_278_MOESM4_ESM.pdf]

COREQ (Consolidated Criteria for Reporting Qualitative Research) – 32 Item Checklist

- ☑ 1. Interviewer/facilitator: Which author conducted the interview or focus group?
- ☑ 2. Credentials: What were the researcher's credentials? (e.g., PhD, MD)
- ☑ 3. Occupation: What was their occupation at the time of the study?
- ☑ 4. Gender: Was the researcher male or female?
- ☑ 5. Experience and training: What experience or training did the researcher have?
- ☑ 6. Relationship established: Was a relationship established prior to study commencement?
- ☑ 7. Participant knowledge of the interviewer: What did the participants know about the researcher?
- ☑ 8. Interviewer characteristics: What characteristics were reported about the interviewer/facilitator ?
- ☑ 9. Methodological orientation and theory: What methodological orientation underpinned the study?
- ☑ 10. Sampling: How were participants selected?
- ☑ 11. Method of approach: How were participants approached?
- ☑ 12. Sample size: How many participants were included in the study?
- ☑ 13. Non-participation: How many people refused to participate or dropped out?
- ☑ 14. Setting of data collection: Where was the data collected?
- ☑ 15. Presence of non-participants: Was anyone else present besides the participants and researchers?
- ☑ 16. Description of sample: What are the important characteristics of the sample?
- ☑ 17. Interview guide: Were questions, prompts, or guides provided by the authors?
- ☑ 18. Repeat interviews: Were repeat interviews carried out?
- ☑ 19. Audio/visual recording: Did the research use audio or visual recording to collect the data?
- ☑ 20. Field notes: Were field notes made during and/or after the interview or focus group?
- ☑ 21. Duration: What was the duration of the interviews or focus groups?
- ☑ 22. Data saturation: Was data saturation discussed?
- ☑ 23. Transcripts returned: Were transcripts returned to participants for comment and/or correction?
- ☑ 24. Number of data coders: How many data coders coded the data?
- ☑ 25. Description of the coding tree: Did authors provide a description of the coding tree?
- ☑ 26. Derivation of themes: Were themes identified in advance or derived from the data?
- ☑ 27. Software: What software, if applicable, was used to manage the data?
- ☐ 28. Participant checking: Did participants provide feedback on the findings?
- ☑ 29. Quotations presented: Were participant quotations presented to illustrate the themes?
- ☑ 30. Data and findings consistent: Was there consistency between the data presented and the findings?
- ☑ 31. Clarity of major themes: Were major themes clearly presented in the findings?
- ☑ 32. Clarity of minor themes: Is there a description of diverse cases or discussion of minor themes?
